# Essential Oil Formulations From *Salvia officinalis*, *Ormenis africana*, and *Mentha pulegium*: Chemical Profiles and Insecticidal Activity Against *Myzus persicae*

**DOI:** 10.1002/fsn3.72024

**Published:** 2026-06-21

**Authors:** Asma El Ayeb‐Zakhama, Roberta Ascrizzi, Hassiba Chahdoura, Muhanad Alhujaily, Guido Flamini, Saad H. Alotaibi, Fethia Harzallah‐Skhiri, Wissem Mnif, Asma Laarif, Ikbal Chaieb

**Affiliations:** ^1^ Production and Protection for Sustainable Horticultural (2PHD) Regional Research Centre in Horticulture and Organic Agriculture, Tunisian Centre‐East Chott‐Mariem Sousse Tunisia; ^2^ Laboratory of “Bioressources: Biology Integrative & Valorisation” (LR14‐ES06) High Institute of Biotechnology of Monastir University of Monastir Monastir Tunisia; ^3^ Dipartimento di Farmacia Università di Pisa Pisa Italy; ^4^ Unite de Recherche UR17ES30 “Génomique, Biotechnologie et Stratégies 8 Antivirales”, Institut Supérieur de Biotechnologie Université de Monastir Monastir Tunisie; ^5^ Institut National des Technologies et des Sciences du Kef Université de Jendouba Kef Tunisie; ^6^ Department of Biochemistry, College of Medicine Imam Mohammad Ibn Saud Islamic University (IMSIU) Riyadh Saudi Arabia; ^7^ Centro Interdipartimentale di Ricerca ‘Nutraceutica e Alimentazione per la Salute’ Nutrafood University of Pisa Pisa Italy; ^8^ Department of Chemistry, Turabah University College Taif University Taif Saudi Arabia; ^9^ Department of Chemistry, College of Science University of Bisha Bisha Saudi Arabia

**Keywords:** bio‐pesticide, essential oils, formulation, greenhouse, laboratory bioassays, *Mentha pulegium*, *Myzus persicae*, *Ormenis africana*, phytotoxicity, *Salvia officinallis*

## Abstract

This study evaluates the yields, chemical compositions, and insecticidal potential of essential oils from *Salvia officinalis* L. (So), *Ormenis africana* (Oa), and *Mentha pulegium* L. (Mp), as well as their mixtures (M_So+Oa_, M_So+Mp_, M_Oa+Mp_, M_So+Oa+Mp_). The essential oil yields were 0.39%, 0.35%, and 0.58% for So, Oa, and Mp, respectively. Chemical analyses showed that So and Mp oils were dominated by oxygenated monoterpenes (camphor (21.9%), α‐thujone (21.6%), 1,8‐cineole (12.5%) in So; pulegone (80.6%) in Mp), while Oa contained oxygenated monoterpenes (artemisia ketone, 23.6%) and oxygenated sesquiterpenes (α‐bisabolol, 28.8%). Mixtures of these oils were rich in oxygenated monoterpenes and oxygenated sesquiterpenes. Laboratory fumigation tests of formulations prepared from individual oils and mixtures revealed that F_So+Oa+Mp_ was the most effective, with an LC_50_ of 31.1 μL/L air, compared to LC_50_ values of 41.53, 84.02, and 32.61 μL/L air for F_So_, F_Oa_, and F_Mp_, respectively. Greenhouse trials confirmed efficacy: fumigation at 4% and 8% caused 30.4% and 65.83% mortality without phytotoxicity, while spraying achieved higher mortality (58.01% and 99.66%) but induced plant damage. This study is the first to evaluate, under greenhouse conditions, formulations based on these essential oils and their mixtures, highlighting synergistic effects and their potential as natural alternatives to synthetic pesticides, offering a promising strategy for eco‐friendly pest management and sustainable agriculture.

## Graphical abstract

The insecticidal potential of formulations based on *Salvia officinalis* , *Ormenis africana*, and *Mentha pulegium* essential oils was investigated against *Myzus persicae* . The combination of the three oils provided the most effective control, showing strong activity in laboratory assays and greenhouse trials. Fumigation effectively reduced aphid populations without phytotoxic effects, whereas spray treatments produced higher mortality but affected plant tissues. The results support the use of essential oil mixtures as promising botanical alternatives for sustainable aphid control.

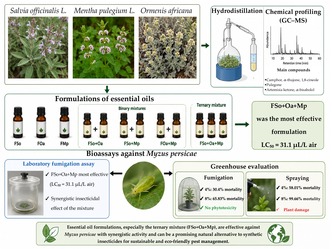

## 1 Introduction

The global human population is expected to reach nearly 9.15 billion by 2050 and 10.4 billion by 2100, and therefore food production must significantly increase (Alexandratos and Bruinsma 2012; Bahar et al. 2020; Dyvik 2024). The rising global food demand will lead necessary to increased dramatically reliance on the use of agrochemicals such as fungicides, insecticides, and herbicides. However, the excessive use of agrochemicals represents a major obstacle to achieving global agricultural security. In fact, their excessive application has led to various undesirable effects, including environmental contamination, development of pesticide‐resistant pest populations, and detrimental impacts on non‐target organisms, including beneficial insects (Devi et al. 2022; Cech et al. 2023; Ren et al. 2023; Daraban et al. 2023; Khan et al. 2023; Ahmad et al. 2024; Barathi et al. 2024; Kaur et al. 2024). It is also well documented that these products present a significant public health problem. Individuals exposed to these toxic substances may develop serious health issues, such as cancer, congenital malformations, neurological disorders, infertility and compromised immune function (Fang et al. 2020; Daraban et al. 2023; Devi et al. 2022; Ahmad et al. 2024; Kaur et al. 2024). In recent years, there has been growing interest in exploring natural alternatives for pest control in agriculture, driven by concerns over the environmental impact and health risks associated with synthetic chemical pesticides (Zarrad et al. 2017; Chaieb et al. 2018; Chowdhury et al. 2024; Daraban et al. 2023). Essential oils, derived from various plant sources gained attention and have emerged as promising alternatives for insect pest control due to their natural insecticidal properties and perceived safety for humans and the environment (Mohan et al. 2011; Ferraz et al. 2022). The insecticidal activity of essential oils has been extensively studied, with research focusing on identifying effective formulations and elucidating their modes of action against target pests (Giuliano et al. 2024; Worku et al. 2024). Essential oils contain a complex mixture of volatile compounds, including terpenoids, phenolics, and aldehydes, which exert insecticidal effects through various mechanisms such as disrupting the insect nervous system, interfering with feeding behavior, and exerting repellent or antifeedant effects (Zhang et al. 2015; Espinoza et al. 2021; Amokrane et al. 2024; Guimarães et al. 2024; Czerniewicz et al. 2024). Individual essential oils have shown insecticidal activity against specific pest species, while formulations comprising mixture of essential oils extracted from different plant species have garnered attention for their potential synergistic or additive effects, which may enhance their efficacy and broaden the spectrum of pest control (Jerbi et al. 2021a, 2021b; Zhu et al. 2024). In greenhouse settings, where plant health and productivity are paramount, biopesticides can play a crucial role in integrated pest management (IPM) strategies. They not only target specific pests but also promote biodiversity and ecological balance, thereby contributing to long‐term sustainability (Baker et al. 2020; Devi et al. 2022). *Myzus persicae* (Sulzer) (Hemiptera: Aphididae), commonly known as the green peach aphid, is a significant pest in agriculture, especially affecting plants from the Solanaceae family (such as peppers and tomatoes) and many other greenhouse crops (Ali 2023). It feeds exclusively on the phloem sap of plants using its piercing‐sucking mouthparts. This feeding method causes both direct and indirect damage to host plants (Capinera 2020). In addition, aphids are especially formidable for their ability to transmit viruses. They are capable of transmitting more than 100 different plant viruses. For example, Potato virus Y (PVY) can be transmitted to tomatoes ( *Solanum lycopersicum* L.), potatoes ( *Solanum tuberosum* L.) and tobacco ( *Nicotiana tabacum* L.) by several species including *M. persicae* (Ali 2023). In this work, this insect was selected for its global predominance, its capacity for virus transmission and its ability to develop resistance to conventional pesticides.

Therefore, this study aimed to investigate the potential use of formulations based on the essential oils of So, Oa, Mp, and their mixtures as biopesticides. Their effectiveness in controlling *M. persicae* was evaluated under both laboratory and greenhouse conditions for the first time.

## 2 Material and Methods

### 2.1 Plant Material

The aerial parts of three aromatic plants: *S. officinalis* , *Ormenis africana*, and *M. pulegium* were collected from plant populations cultivated at an experimental field in the Regional Center of Horticulture and Organic Agriculture Research of Chott‐Meriem (CRRHAB), a coastal region in Tunisia (35°56′17″ N, 10°33′18″ E). Voucher specimens (No. 20So; 21Oa; 22Mp) were deposited in the Herbarium of the Laboratory of Genetic Biodiversity and Valorisation of Bioresources, High Institute of Biotechnology of Monastir, Tunisia.

### 2.2 Essential Oil Extraction

Each fresh plant material (4 × 250 g) was cut into small pieces and submitted to hydrodistillation for 4 h using a *Clevenger*‐type apparatus (flask capacity 1000 mL, model TF‐1000 mL; TEFIC BIOTECH CO., Xi'an, PR China). The obtained oils were weighed, the yield of each sample was calculated and expressed in % (*v/w*) with respect to fresh material. All the oils were stored in dark glass vials at 4° until analysis. All the extractions were done in triplicate.

### 2.3 GC Analysis

The essential oils were submitted to GC‐FID and GC/MS analyses. The gas chromatography analysis was performed using a Hewlett‐Packard 5890 apparatus equipped with a flame ionization detector (FID) and an HP‐5 capillary column (30 m × 0.25 mm i.d., film thickness 0.25 μm). The oven temperature was initially set isothermal at 50°C for 1 min, then increased from 50°C to 280°C at a rate of 5°C per minute, followed by a hold at 280°C for an additional 3 min. The injector temperature was maintained at 250°C, while the detector temperature was set at 280°C. Nitrogen was used as the carrier gas at a flow rate of 1.2 mL/min. A sample volume of 1 μL, consisting of a 10% essential oil solution in hexane, was injected for analysis. Component identification was achieved by comparing their retention times with those of pure authentic samples and utilizing Linear Retention Indices (L.R.I.) relative to a series of n‐hydrocarbons ranging from C9 to C28.

The essential oils were analyzed using a Varian CP‐3800 gas chromatography apparatus coupled with a Varian Saturn 2000 ion trap mass detector, utilizing an HP‐5 capillary column measuring 30 m in length, with a 0.25 mm internal diameter and a film thickness of 0.25 μm. Helium served as the carrier gas at a flow rate of 1 mL/min. The operational parameters for the mass spectrometer included an ionization voltage set at 70 eV, with injector and transfer line temperatures maintained at 220°C and 240°C, respectively. The oven temperature was programmed to increase from an initial 60°C to a final temperature of 240°C at a rate of 3°C per minute. A sample volume of 1 μL, prepared as a 10% solution in hexane, was injected for analysis.

### 2.4 Compound Identification

The identification of the constituents was based on comparison of their retention times (*t*R) with those of authentic samples, comparing their linear retention indices (*LRIs*) relative to the series of *n*‐hydrocarbons and on computer matching against commercial (NIST 98 and ADAMS) and home‐made library mass spectra built up from pure substances and components of known oils and MS literature data (Joulain and Koenig 1998; Adams 2007). Moreover, the molecular weights of all the identified substances were confirmed by *GC/CI‐MS*, using MeOH as *CI* ionizing gas.

### 2.5 Preparation of Essential Oil Mixtures and Different Formulations

After the extraction of three essential oils, different mixtures were prepared as follows: M_So+Oa_ (50%_So_ + 50%_Oa_), M_So+Mp_(50%_So_ + 50%_Mp_), M_Oa+Mp_(50%_Oa_ + 50%_Mp_) and M_So+Oa+Mp_ (33.33%_So_ + 33.33%_Oa_ + 33.33%_Mp_). Seven liquid formulations were prepared by dispersing 1%, 2%, 4% and 8% of individual essential oils or their mixtures in an aqueous solution containing 2% Tween 80 as an emulsifying agent. In addition, TRACER (a commercial insecticide) was prepared in an aqueous solution containing 2% of Tween 80 and used us the positive control (+), An aqueous solution containing 2% of Tween 80 served as the negative control (−). In total, seven formulations were obtained: F_So_, F_Oa_, F_Mp_, F_So+Oa_ F_So+Mp_, F_Oa+Mp_, F_So+Oa+Mp_.

### 2.6 Rearing of the Insect *Myzus percicae* for In Vitro Testing

For in vitro testing of the insecticidal activity of the different formulations, sweet pepper ( *Capsicum annuum* L., Solanaceae), a host plant highly favorable for aphid development, was used for insect rearing. Seedlings of sweet pepper developed at the 3–4 leaf stage in pots were placed in wooden cages covered by a mosquito netting to prevent contamination by other insect species. The cages were maintained in a greenhouse to promote aphid survival and multiplication in favorable conditions. The plants were irrigated regularly to ensure optimal growth. The aphid species used in this study was *Myzus persicae* (Sulzer) (Hemiptera: Aphididae). After installing the cages, a small number of *M. persicae* were introduced onto the leaves of sweet pepper plants to initiate colony establishment. The aphid population increased progressively over the following days, resulting in full infestation of the plants. The initial population of *M. persicae* was collected from a greenhouse‐grown chili pepper crop in the Chott Mariem region (Tunisia) and morphologically identified prior to colony establishment. A continuous laboratory colony was subsequently maintained on chili pepper plants prepared specifically for rearing. To ensure age synchronization, wingless adult females were periodically transferred to fresh host plants. Aphid density was carefully regulated and maintained below 50 individuals per leaf to prevent overcrowding.

### 2.7 Insecticidal Activity Under Laboratory Conditions

The insecticidal activity of the different formulations was evaluated in vitro by fumigation tests then the most effective formulation will be tested in vivo by fumigation and spray methods. The effectiveness of these formulations was determined by examining the mortality rates of *M. persicae* . For in vitro bioassays, only newly molted apterous adult females were selected. All manipulations were performed gently using a fine brush to minimize stress and physical damage to the insects. Indeed, Whatman N°1 filter papers, each with a diameter of 2 cm, were impregnated with different formulations at concentrations of 1, 2, 4, and 8 of the EOs and their mixtures to release fumigant. Each impregnated filter paper was attached to the screw cap of a 40 mL Plexiglas bottle. Ten adults of *M. persicae* were added to each bottle and caps were screwed on tightly. Each treatment and control was replicated five times. After exposure period, the number of dead and alive insects in each bottle was counted to assess the efficacy of the formulations.

### 2.8 Insecticidal Activity Under Greenhouse Conditions

The effectiveness of the formulation F_So+Oa+Mp_ was assessed in a greenhouse pepper cultivation setup at the CRRHAB experimental station. No insecticide treatments were utilized during this evaluation. The greenhouse maintained optimal conditions for plant growth: temperature ranging between 22°C–28°C, relative humidity of 60%–80%, and a photoperiod of 12 h of light and 12 h of darkness. The experimental greenhouse was divided into eight compartments, each measuring 1 m^3^, which were separated by plastic barriers to isolate the treatments. The first compartment received a treatment with a liquid formulation F_So+Oa+Mp_ containing 4% EOs, while the second compartment was treated with the same formulation containing 8% EOs. The third compartment was treated with TRACER commercial insecticide as a positive control and the fourth compartment served as a negative control using a formulation without EOs. These last four compartments were used for the fumigation test (indirect application through vaporization). The other four compartments maintained under the same conditions with the same formulations were intended for foliar spray treatment (applied directly to the plants). For the tests we chose eight plants randomly per compartment. Ten leaves were marked on each plant for monitoring purposes. Adult insects present on the upper and lower leaf surfaces were counted visually by direct observation. Counts were conducted during the early morning hours (08:00–10:00 h) when insect activity was relatively low, minimizing movement and potential recounting errors. In cases where pest density was high, a hand lens (10× magnification) was used to ensure accurate identification and counting. Only newly molted apterous female adult individuals were recorded. This ensured uniformity in life stage composition across all treatments at baseline. Counts were performed 24 h after treatment on the same marked plants. For each observed plant, the total numbers of live and dead adult insects were recorded on the previously marked leaves. The mortality rates of the pests were assessed. The purpose of calculating this mortality rate was to determine the efficacy of the formulation at different concentrations in controlling pest populations compared to chemical control and untreated plants.

### 2.9 Statistical Analyses

Values were expressed as average of five replicates. The variance analysis was done by one‐way ANOVA at *p* < 0.05. Comparisons of averages were performed by Duncan Multiple Range test using version 18 of the Statistical Package for the Social Sciences program (SPSS). Lethal Concentrations (LC_50_) were calculated based on the obtained results of insecticidal effect. LC_50_ values were calculated using probit analysis. The combined effect of essential oil mixtures was determined on the basis of LC_50_ by using the synergistic ratio (SR) model (Hewlett and Plackett 1959):

$$\mathrm{SR}={\mathrm{LC}}_{50}\;\left(\text{essential oil alone}\right)/{\mathrm{LC}}_{50}\;\left(\text{mixture}\right)$$

where SR is 1 for additive effect, SR < 1 for antagonistic effect and SR > 1 for synergistic effect.

## 3 Results and Discussion

### 3.1 Yields of the Essential Oils

The oil yields varied between 0.35%–0.58%. The lowest oil percentages were recorded in *Ormenis africana* and *Salvia officinalis* (0.35 and 0.39%, resp.), the highest in *Mentha pulegium* (0.58%).

### 3.2 Essential Oils Compositions

Chromatographic analysis (GC‐FID and GC/MS) of the essential oils allowed the identification of 40 compounds (Table 1), representing 98.4%–99.8% of the total oils composition.

**TABLE 1:** Chemical composition of the essential oils extracted from the aerial parts of *Salvia officinalis* (So), *Ormenis africana* (Oa) and *Mentha pulegium* (Mp) cultivated in Tunisia and their mixtures M_So+Oa_, M_So+Mp_, M_Oa+Mp_, M_So+Oa+Mp_.

No.	Compound name and class	*R.I* ^a^	Content [%]^b^						
			So	Oa	Mp	M_So+Oa_	M_So+Mp_	M_Oa+Mp_	M_So+Oa+Mp_
1	Santolina triene	911	—	0.7	—	0.3	—	0.3	—
2	*α*‐Pinene	941	0.7	0.4	0.3	0.5	0.5	0.3	0.4
3	Camphene	955	1.7	2.0	0.1	1.6	0.9	0.8	1.1
4	Sabinene	977	—	2.1	0.2	0.9	—	0.9	0.6
5	*β*‐Pinene	982	1.0	3.0	0.4	1.7	0.8	1.3	1.5
6	Myrcene	993	0.5	4.4	0.1	2.2	0.3	1.7	1.4
7	Yomogi alcohol	1001	—	0.6	—	0.3	—	0.2	—
*8*	*p*‐Cymene	1028	0.6	—	—	0.4	0.4	—	0.3
9	Limonene	1032	1.0	—	0.3	—	0.7	—	—
10	*β*‐Phellandrene	1033	—	10.3	—	5.0	—	3.9	2.9
11	1,8‐Cineole	1034	12.5	—	3.5	6.6	8.3	2.2	6
12	*γ*‐Terpinene	1062	0.2	—	—	—	—	—	—
13	Artemisia ketone	1063	—	23.6	—	10.9	—	9.4	6.4
14	Terpinolene	1090	0.1	1.8	—	1.0	—	0.5	0.4
15	*α*‐Thujone	1105	21.6	—	—	12.0	10.4	—	7.5
16	*β*‐Thujone	1118	4.6	—	—	2.6	2.2	—	1.6
17	Camphor	1145	21.9	12.9	0.2	18.2	10.6	5.3	11.1
18	Menthone	1155	—	—	1.1	—	0.6	0.7	0.5
19	Isomenthone	1166	—	—	0.8	—	0.4	0.4	0.4
20	Borneol	1167	1.3	0.5	1.0	1.3	1.3	0.8	1.1
21	Isopulegone	1174	—	—	1.3	—	0.5	0.7	0.4
22	4‐Terpineol	1179	0.9	1.0	—	1.0	0.5	0.4	0.6
23	Cryptone	1185	—	1.1	—	—	—	1.1	0.5
24	α‐Terpineol	1191	0.3	—	0.6	—	0.4	0.4	0.3
25	Verbenone	1205	—	—	0.3	—	—	—	—
26	Pulegone	1239	0.3	—	80.6	—	45.0	50.5	34.2
27	Bornyl acetate	1287	0.4	—	—	—	—	—	—
*28*	*p*‐Cymen‐7‐ol	1290	—	—	1.4	—	—	—	—
29	Piperitenone	1342	—	—	4.1	—	2.0	2.3	1.5
30	*β*‐Caryophyllene	1419	2.5	—	0.4	1.6	1.1	0.4	1.1
31	*α*‐Humulene	1455	1.6	—	—	0.9	0.6	—	0.6
32	Germacrene D	1482	—	3.6	—	1.8	—	1.2	0.8
33	*δ*‐Cadinene	1524	0.4	—	—	—	—	—	—
*34*	*(E)*‐Nerolidol	1564	—	1.4	—	—	—	0.5	0.3
35	Caryophyllene oxide	1581	2.6	0.8	—	2.0	1.5	—	1.1
36	Viridiflorol	1591	11.0	—	—	6.7	4.8	—	3.4
37	Humulene epoxide II	1607	1.6	—	—	1.0	0.8	—	0.6
38	*T*‐Cadinol	1641	—		1.7		0.8	1.4	0.9
39	*α*‐Bisabolol	1685	—	28.8	—	14.5	—	11.9	7.5
40	Manool	2056	10.5	—	—	4.6	4.1	—	2.7
	Monoterpene hydrocarbons		5.8	24.7	1.4	13.6	3.6	9.7	8.6
	Oxygenated monoterpenes		63.8	38.6	94.9	52.9	82.2	73.3	71.6
	Sesquiterpene hydrocarbons		4.5	3.6	0.4	4.3	1.7	1.6	2.5
	Oxygenated sesquiterpenes		15.2	31.0	1.7	24.2	7.9	13.8	13.8
	Apocarotenes		10.5	0.0	0.0	4.6	4.1	0.0	2.7
	Non‐terpene derivatives		0.0	1.1	0.0	0.0	0.0	1.1	0.5
	Total identified (%)		99.8	99.0	98.4	99.6	99.5	99.5	99.7

*Note:* —, not detected.

^a^ *RI*: retention index determined rel. to the series of n‐alkanes (C_9_–C_28_) on a HP‐5 capillary column.

^b^ Content determined on a HP‐5 capillary column.

### 3.3 *Salvia officinalis* Oil Composition

In total, 24 compounds were identified in the *S. officinalis* oil representing 99.8% of the total volatiles (Table 1). The main constituents belong to oxygenated monoterpenes class (63.8%). Camphor (**17**; 21.9%), *α*‐thujone (**15**; 21.6), 1,8‐cineole (**11**; 12.5%), and *β*‐thujone (**16**; 4.6%) were the major ones. Viridiflorol (**36**; 11%) was the main component of oxygenated sesquiterpenes class (15.2%).

These results are consistent with those reported by Ben Khedher et al. (2017), who identified 49 components in the essential oil of *S. officinalis* leaves. In their study, the major constituents were camphor (25.14%), *α*‐thujone (18.83%), 1,8‐cineole (14.14%), viridiflorol (7.98%), *β*‐thujone (4.46%), and *β*‐caryophyllene (3.30%). Our findings are in strong agreement with previous reports indicating that oxygenated monoterpenes represent the predominant chemical class in *S. officinalis* essential oil, exceeding the proportions of other compound classes (Rguez et al. 2013; Kammoun El‐Euch et al. 2019; Fellah et al. 2006; Tundis et al. 2020; Jažo et al. 2023). This predominance of oxygenated monoterpenes may explain the biological activities commonly attributed to this species.

### 3.4 Ormenis Africana Oil Composition

In total, 18 compounds were identified in the *O. africana* oil corresponding to 99% of the total essential oil (Table 1). The most abundant of them were *α*‐bisabolol (**39**; 28.8%), artemisia ketone (**13**; 23.6%), followed by camphor (**17**; 12.9%), *β*‐phellandrene (**10**; 10.3%), and myrcene (**6**; 4.4%). The EO of *O. africana* was composed of 38.6% of compounds belonging to the oxygenated monoterpenes class, 31% of them to the oxygenated sesquiterpenes class, and 24.7% to monoterpene hydrocarbons. The other classes are in weak percentages (0%–3.6%). Indeed, our results are consistent with those reported by Bel Hadj Salah‐Fatnassi et al. (2017), who demonstrated that the essential oil was mainly characterized by two predominant classes: oxygenated monoterpenes (36.94%) and oxygenated sesquiterpenes (29.8%). The latter were primarily represented by *β*‐eudesmol (10.9%) and spathulenol (5.8%). Similarly, Alves‐Silva et al. (2019) reported that *O. africana* essential oil was predominantly composed of monoterpenes, with *β*‐pinene (22.5%), 1,8‐cineole (10.0%), limonene (9.1%), camphor (8.1%), and *β*‐phellandrene (8.0%) identified as the principal constituents. In the same context, Lmachraa et al. (2013) found that oxygenated monoterpenes constituted the majority of the chemical composition (79.24%), with camphor (54.30%) as the dominant compound, followed by borneol (17.24%) and 1,8‐cineole (5.27%).

### 3.5 *Mentha pulegium* Oil Composition

In *M. pulegium* oil, 19 compounds were identified (Table 1). The major constituent was pulegone, an oxygenated monoterpene (**26**; 80.6%). Pulegone is widely recognized as the characteristic chemotype of *M. pulegium* . Several studies investigating the chemical composition of *M. pulegium* essential oil, both in Tunisia and in other countries, have consistently reported pulegone as the predominant compound (Marzouk et al. 2008; Boutabia et al. 2020; Ez‐Zriouli et al. 2022; Laftouhi et al. 2023). These findings further confirm the stability of the pulegone chemotype in this species, although quantitative variations may occur depending on geographical origin, environmental factors, and extraction conditions.

### 3.6 Mixtures Oil Composition

Analysis of the M_So+Oa_ EO mixture shows a total of 25 constituents corresponding to 99.6% of the total essential oil (Table 1). Indeed, camphor (**17**; 18.2%), *α*‐bisabolol (**39**; 14.5%) and *α*‐thujone (**15**; 12%) are the main compounds. They belong to the classes of oxygenated monoterpenes and oxygenated sesquiterpenes. A total of 25 constituents were identified for F_So+Mp_ mixture representing 99.5% of the total oil. Oxygenated monoterpenes are the dominant group of compounds, mainly including pulegone (**26**; 45%), camphor (**17**; 10.6%) and *α*‐thujone (**15**; 10.4%). For the F_Oa+Mp_ mixture, a total of 26 constituents were identified, representing 99.5% of the total oil. Oxygenated monoterpenes compounds were dominant (73.3%), followed by oxygenated sesquiterpenes ones (13.8%). The main compounds included pulegone (**26**; 50.5%) and α‐bisabolol (**39**; 11.9%). A total of 32 constituents, representing 99.7% of the composition of M_So+Oa+Mp_ mixture. Were identified. The analysis revealed that this mixture is particularly rich in pulegone (**26**; 34.2%), camphor (**17**; 11.1%) and *α*‐bisabolol (**39**; 7.5%). According to the results of the mixture analyses, the major compounds identified in the individual essential oils were also present in the mixtures.

The GC–MS analysis of the essential oil mixtures revealed a predominantly additive chemical behavior, as the major constituents identified in the individual oils were consistently detected in the corresponding binary and ternary mixtures, indicating a predominantly additive chemical behavior. In the M_So+Oa_ mixture, camphor, *α*‐bisabolol, and *α*‐thujone were the principal compounds, reflecting the combined contribution of oxygenated monoterpenes and oxygenated sesquiterpenes from both oils. Similarly, the F_So+Mp_ and F_Oa+Mp_ mixtures were largely dominated by oxygenated monoterpenes, particularly pulegone, whose high proportion indicates the strong influence of *M. pulegium* essential oil within these mixtures. In the ternary mixture M_(So+Oa+Mp)_, an increase in the total number of identified constituents (32 compounds, 99.7%) was observed, indicating greater chemical diversity resulting from the combination of the three oils. However, the major constituents remained pulegone, camphor, and *α*‐bisabolol, confirming that no new major compounds were formed after blending. The variations in relative percentages among mixtures can be attributed to proportional dilution effects rather than chemical interactions, supporting the additive nature of the volatile profiles. Similar observations have been reported in other studies on essential oil mixtures. For example, El Amrani et al. (2025) demonstrated that the binary and ternary mixtures of *Thymus serpyllum* , *Mentha pulegium* and Mentha piperita maintained the major volatile compounds of the individual oils in mixture. Recent work by Hamdeni et al. (2026) also reported additive and potential synergistic interactions in selected essential oil binary and ternary mixtures when examined by GC–MS and multivariate analyses. Overall, these results demonstrate that combining essential oils increases chemical diversity while maintaining the key bioactive constituents, which may enhance their potential as botanical insecticides.

### 3.7 Fumigation Test in Laboratory

The results of evaluation of the fumigant activity of formulations based on EOs of *M. pulegium* , *S. officinalis* , and *O. africana* are presented in the Table 2. The mortality tests highlight the insecticidal activity of the different formulations. The analysis of the results shows that the insecticidal potential varies depending on both the concentration and the EOs used. At the lowest concentration (1% and 2%), *M. pulegium* EO exhibited the greatest toxic effect, causing mortality rates of 42% and 46%, respectively. At these concentrations, the highest mortality (50%) was observed with the F_Oa+Mp_ formulation. At a concentration of 4%, So and Mp EOs induced mortality rates of 76% and 60%, respectively, whereas the F_So+Oa+Mp_ formulation achieved a mortality of only 60%. At the highest concentration 8%, all formulations exhibited very high toxicity against *M. persicae* (82%–96%), except F_Oa_ which showed a mortality rate of 50%. Regarding the variation within the same mixture, statistical analysis indicated that the most effective formulations were generally observed at concentrations of 4% and 8%. In fact, toxicity increased with increasing essential oil concentration. In addition, based on LC_50_ values, the formulations were classified according to their toxic potency (Table 3). Formulations F_Mp_, M_Oa+Mp_ and F_So+Oa+Mp_ were the most effective., with the lowest LC_50_ values of 32.61 μL/L air, 32.66 μL/L air and 31.10 μL/L air, respectively. In contrast, F_Oa_ exhibited the lowest fumigant activity, with the highest LC_50_ (84.02 μL/Lair). The strong insecticidal effects of *M. pulegium* , *S. officinalis* and *O. africana* essential oils are consistent with previous studies. For instance, Chaieb and Roman (2019) showed that *M. pulegium* EO exhibited excellent efficacy in both contact and fumigation tests against aphids, indicating its potential as an optimal source of active substances for the development of botanical aphicides. Furthermore, Brahmi et al. (2016) and Salem et al. (2017) demonstrated fumigation contact toxicity and repellency properties of *M. pulegium* EO against adults of *Rhizophtera dominica*, *Tribolium castaneum* and *Lasioderma serricorne* . Similarly, Rguez et al. (2013) reported that the essential oil of *S. officinalis* exhibits toxic activity against *Spodoptera littoralis*. In addition, Abdellaoui et al. (2017) showed that *S. officinalis* essential oil possesses both toxic and repellent effects against adults and larvae of *Tribolium confusum* , with median lethal concentrations (LC50) of 0.13 and 0.16 μL/cm^2^ for larvae and adults respectively. Howerver, our results obtained for *O. africana* essential oil differ from those reported in previous. For instance, De Elguea‐Culebras et al. (2017) reported insecticidal activity of EO from *O. africana* against ticks and aphids. Moreover, Suresh et al. (1996) and Czerniewicz and Chrzanowski (2021) demonstrated the toxicity of *O. africana* EO against *Nilaparvata lugens* , *M. persicae* (Sulzer) and *Rhopalosiphum padi* . This fluctuation between our results and those reported in the literature may be attributed in the tests performed. Indeed, in our experimental conditions we used fumigation test while in the other studies the tests were carried out by contact exposure. Furthermore, several EOs have been reported to exhibit toxic effects against aphids, including *Foeniculum vulgare* EO against *M. persicae* (Pavela 2018), *Melaleuca styphelioides* EO against *A. gossypii* , *M. persicae* and *A. spiraecola* (Albouchi et al. 2018). The combination of essential oils may generate additive or synergistic effects, thereby enhancing their insecticidal efficacy. The rationale behind using mixed formulations lies in the variety of bioactive compounds present in different essential oils, allowing them to target multiple physiological pathways in insects. As a result, this strategy not only improves efficacy but also reduce the likelihood of resistance development in insect populations. Several studies have demonstrated the synergistic effects of essential oil mixtures in insecticidal formulations (Skuhrovec et al. 2017; Kim et al. 2018; Lee et al. 2020; Aungtikun et al. 2021).

**TABLE 2:** Percentage of mortality of *Myzus persicae* adults exposed for 24 h to different formulations based on essential oils and their mixtures.

Dose (% of EO)	Mortality (%)						
	F_So_	F_Oa_	F_Mp_	F_So +Oa_	F_So+Mp_	F_Oa+Mp_	F_So+Oa+Mp_
0	0 ± 0.00^Aa^	0 ± 0.00^Aa^	0 ± 0.00^Aa^	0 ± 0.00^Aa^	0 ± 0.00^Aa^	0 ± 0.00^Aa^	0 ± 0.00^Aa^
1	28 ± 0.08*,^Ab^	22 ± 1.00^Bb^	42 ± 1.00^Bb^	12 ± 0.00^Aa^	8 ± 0.00^Aa^	30 ± 2.00^ABb^	32 ± 0.90^Bb^
2	36 ± 0.10^Ab^	26 ± 0.10^Ba^	46 ± 1.00^Bb^	38 ± 0.00^Bb^	28 ± 0.60^Ba^	50 ± 0.50^Bb^	40 ± 0.60^Bb^
4	76 ± 0.09^Bb^	48 ± 0.08^Ca^	60 ± 0.10^Bb^	38 ± 1.00^Bb^	28 ± 0.60^Ba^	50 ± 0.40^Bb^	60 ± 0.00^Bb^
8	82 ± 0.80^Bb^	50 ± 0.00^Ca^	96 ± 0.00^Cb^	90 ± 0.00^Db^	96 ± 0.00^Db^	90 ± 1.00^Cb^	96 ± 2.00^Cb^

*Note:* Within columns, means followed by the same letter (lowercase letters) were not statistically different based on Duncan Multiple Range test at *p* < 0.05. Within rows, means followed by the same letter (uppercase letters) were not statistically different based on Duncan Multiple Range test at *p* < 0.05.

* Values are means of five replications, each set‐up with 10 insects.

**TABLE 3:** LC_50_ values of fumigant toxicity of different essential oils and mixtures against *Myzus Persicae* adults.

EO formulation	F_So_	F_Oa_	F_Mp_	F_Oa+So_	F_So+Mp_	F_Oa+Mp_	F_So+Oa+Mp_
LC_50_	41.53	84.02	32.61	47.19	47.70	32.66	31.10
(μL/L air)	(0.30–122.39)*	(6.02–97.55)	(7.65–69.08)	(33.86–65.76)	(43.23–52.77)	(6.24–66.23)	(13.62–54.99)
*χ* ^2^	28.35	15.58	20.14	08.77	04.36	28.11	16.85
df	3	3	3	3	3	3	3
*p*	< 0.001	< 0.001	< 0.001	< 0.001	< 0.001	< 0.001	< 0.001
Synergistic ratio (SR)	—	—	—	1.780	0.870	2.572	2.701
Effect	—	—	—	Synergistic	Antagonistic	Synergistic	Synergistic

* For LC_50_ units (μL/L air), lower and upper confidence limits are given in parenthesis.

### 3.8 Insecticidal Activity Under Greenhouse Conditions

**FIGURE 1:**
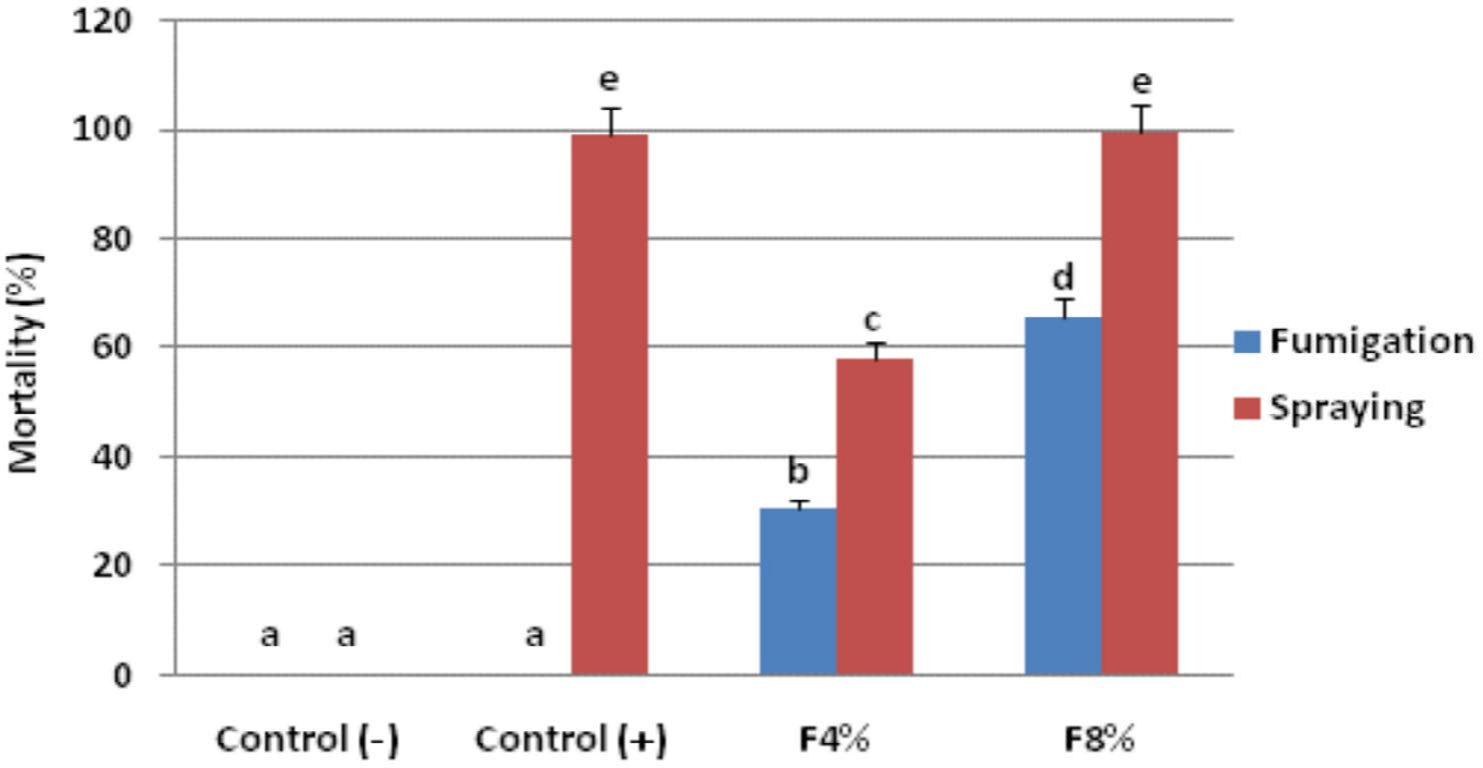
Mortality (%) of *M. persicae* after 24 h exposure to the formulation containing a mixture of three essential oils (F_So+Oa+Mp_) at two concentrations (F4% and F8%) under greenhouse conditions, applied by fumigation and spraying. Control (+) corresponds to TRACER (a commercial insecticide), whereas Control (−) represents the formulation without essential oils. Vertical bars indicate standard error. Different letters indicate significant differences among treatments according to Duncan's multiple range test (*p* ≤ 0.05).

**FIGURE 2:**
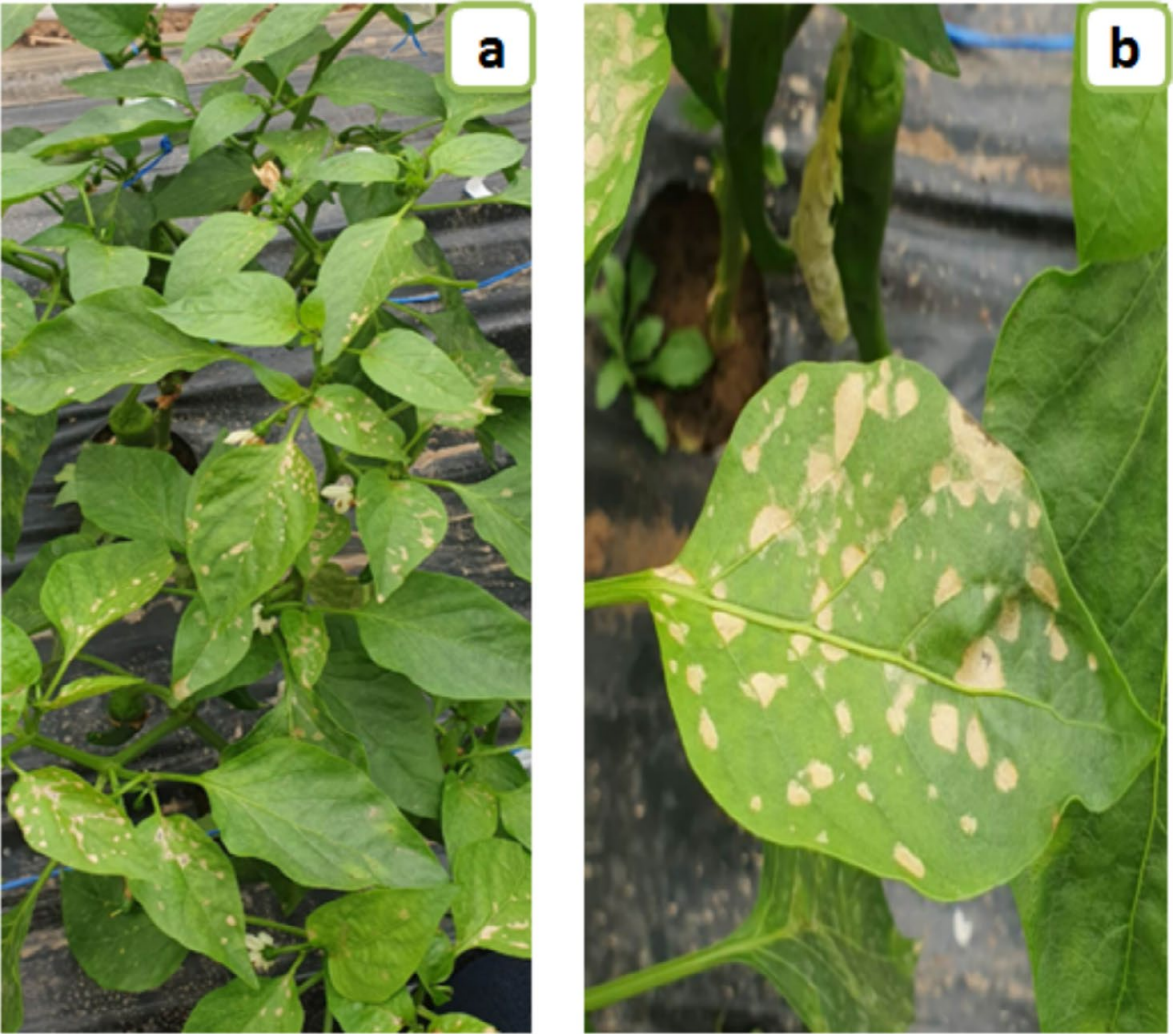
Phytotoxic symptoms on pepper plants after spray application of the formulation based on mixtures of three essential oils F_So+Oa+Mp_ after 24 h: (a) General view of the plant showing numerous chlorotic and necrotic spots on leaves. (b) Detailed view of leaf damage characterized by irregular chlorotic and necrotic lesions.

To address the knowledge gap regarding the insecticidal activity of EOs against *M. persicae* , especially under controlled conditions, the optimized in vitro formulation F_So+Oa+Mp_ was selected for greenhouse evaluation using two different application methods: spraying and fumigation at two concentrations (4% and 8%) (Figure 1). The fumigation application of the F_So+Oa+Mp_ formulation proved to be effective, resulting in mortality rates of *M. persicae* reaching 30.4% and 65.83% at concentrations of 4% and 8%, respectively, after 24 h of exposure. In addition, no phytotoxic effects were observed on the treated plants. Regarding the spraying method, the formulation exhibited a significant insecticidal effect against *M. persicae* , with mortality rates of 58.01% and 99.66% at concentrations of 4% and 8%, respectively. These greenhouse results corroborate the observations obtained under laboratory conditions. However, when applied by spraying, the formulation induced phytotoxic effects on pepper plants at both concentrations (Figure 2). This finding highlights a critical limitation of the spraying approach, suggesting that careful optimization of concentration or formulation is required to avoid plant damage. Advanced formulation strategies, such as microencapsulation and nanoformulation, have been reported to reduce the phytotoxic effects and prolong the bioactivity of essential oils used as pesticides (De Oliveria et al. 2014; Nebapure et al. 2022).

The strong insecticidal activity of the formulation F_So+Oa+Mp_ is likely attributable to its high content of bioactive compounds, particularly pulegone, camphor, and α‐bisabolol. The additive chemical behavior of the essential oil mixture ensures that these key constituents are preserved in the combined formulation, providing a plausible explanation for its efficacy. These findings are consistent with previous studies reporting that essential oils exert greater insecticidal effects when applied via direct contact, whereas fumigation offers effective pest control with reduced risk of phytotoxicity to the host plant (Chaieb and Roman 2019; Werrie et al. 2020; Ahmadi et al. 2022; Ren et al. 2022; Popescu et al. 2024).

Overall, these results indicate that the F_So+Oa+Mp_ mixture is a potent botanical insecticide against *M. persicae* . However, optimizing application methods and exploring advanced formulation strategies, such as microencapsulation or nanoformulation, will be essential to maximize pest control efficacy while minimizing potential damage to crops.

The present study further demonstrates the efficacy of the combined F_So+Oa+Mp_ formulation against aphid populations in greenhouse‐grown pepper plants, representing a novel approach to integrating multiple essential oils for pest management. While numerous studies have investigated the insecticidal properties of individual essential oils, the potential synergistic effect of these three oils within a single formulation has not previously been reported, highlighting the originality of this approach. The observed mortality rates obtained through both foliar spraying and fumigation suggest that this formulation could serve as a promising alternative to synthetic insecticides, in line with the growing interest in environmentally friendly pest management strategies.

From a food safety perspective, it is important to consider that certain constituents of the essential oil, such as camphor and pulegone, are subject to regulatory limits due to their potential toxicity at high concentrations. Although residue analysis was not within the scope of the present study, the application method, dosage, and pre‐harvest interval are expected to influence residue levels on treated crops. Therefore, further studies focusing on residue quantification and compliance with established safety standards are necessary before practical application on food crops.

## 4 Conclusion

In conclusion, the present study demonstrates that the optimized F_So+Oa+Mp_ essential oil formulation exhibits strong insecticidal activity against *M. persicae* under greenhouse conditions, highlighting its potential as a sustainable botanical alternative to synthetic insecticides. The observed efficacy is likely related to synergistic interactions among the major bioactive constituents of the combined oils. These findings are particularly relevant to the fields of Food Science and Nutrition, as the use of plant‐derived bioinsecticides may contribute to reducing synthetic pesticide residues on food crops, thereby improving the safety and quality of fresh produce such as peppers. Consequently, essential oil–based formulations represent a promising approach for environmentally friendly pest management and safer food production. Further research focusing on formulation optimization and application strategies will be necessary to facilitate their integration into sustainable agricultural systems and integrated pest management programs.

## Author Contributions

**Asma El Ayeb‐Zakhama:** conceptualization, methodology, formal analysis, investigation, resources, writing – original draft. **Asma Laarif:** conceptualization, methodology, formal analysis, investigation, writing – original draft, data curation. **Saad H. Alotaibi:** formal analysis, writing – review and editing, investigation, software. **Fethia Harzallah‐Skhiri:** data curation, formal analysis, supervision, visualization, investigation. **Hassiba Chahdoura:** methodology, formal analysis, writing – original draft, software, investigation. **Roberta Ascrizzi:** methodology, formal analysis, validation, supervision, writing – review and editing. **Muhanad Alhujaily:** formal analysis, writing – review and editing, funding acquisition, supervision. **Guido Flamini:** methodology, formal analysis, validation, investigation, writing – review and editing. **Ikbal Chaieb:** conceptualization, methodology, writing – original draft, investigation, resources, formal analysis, data curation, project administration. **Wissem Mnif:** formal analysis, writing – review and editing, validation, funding acquisition, project administration.

## Conflicts of Interest

The authors declare no conflicts of interest.

## Data Availability

The results presented in this study are accessible on request from the corresponding authors.
